# Pre-synchronization with GnRH or hCG enhances fertility outcomes of heat synch protocol in Holstein dairy cows across parities: A pilot study

**DOI:** 10.1016/j.vas.2026.100739

**Published:** 2026-06-16

**Authors:** Pouya Navidifar, Amirali kaveh, Seyed Edris Hosseini, Hasan keshtkar zolghadr, Roxana Simiyari, Saeed Taghinasab, Mohammad Saied Salehi, Ali Kahyani

**Affiliations:** a Department of Clinical Sciences, Faculty of Veterinary Medicine, Tabriz Medical Sciences Branch, Islamic Azad University, Tabriz, Iran; b Department of Clinical Sciences, School of Veterinary Medicine, Shiraz University, Shiraz, Iran; c Department of Animal Sciences, School of Agriculture, Shiraz University, Shiraz, 71441–65186, Iran

**Keywords:** Heat synch, Pre-synchronization, GnRH, hCG, Fertility, Holstein dairy cows

## Abstract

Efficient estrus synchronization is important for improving reproductive performance in dairy herds; however, variations in ovarian activity among cows of different parities may influence responses to synchronization protocols. The present study evaluated the effects of pre-synchronization with gonadotropin-releasing hormone (GnRH) or human chorionic gonadotropin (hCG) before the Heat Synch protocol on estrus expression, pregnancy outcomes, and serum progesterone concentrations in Holstein cows of different parities. The Heat Synch protocol consisted of GnRH administration on Day 0, prostaglandin F₂α (PGF₂α) administration on Days 7 and 8, and estradiol benzoate administration on Day 9 before insemination. A total of 294 Holstein cows were allocated to nine groups: Heat Synch (HS) alone, GnRH+HS, or hCG+HS in primiparous (n = 99), second-parity (n = 99), and multiparous (n = 96) cows. Estrus detection was followed by artificial insemination according to the AM–PM rule, whereas cows that failed to exhibit estrus underwent fixed-time artificial insemination. Pregnancy was diagnosed by ultrasonography on Days 30 and 60 post-insemination. Serum progesterone concentrations were evaluated at the beginning of the study, before PGF₂α administration, and before estradiol benzoate administration using ELISA. Pre-synchronization with GnRH or hCG significantly increased estrus expression compared with the HS protocol alone. The hCG groups showed higher progesterone concentrations, and no statistically significant parity-related differences in reproductive responses to the synchronization protocols were detected under the conditions of this study. These findings suggest that GnRH- or hCG-based pre-synchronization before Heat Synch may improve reproductive performance under practical dairy farm conditions while promoting behavioral estrus expression before insemination. However, the relatively limited sample size within some subgroups should be considered when interpreting parity-related comparisons.

## Highlights

• Pre-synchronization with GnRH or hCG improved estrus expression in Heat Synch protocols.
• hCG pre-synchronization was associated with higher progesterone concentrations.
• Comparable reproductive responses to pre-synchronization protocols were observed across parity groups.

## 1 Introduction

Efficient reproductive performance is a key determinant of profitability in dairy herds. To maintain optimal productivity, dairy cows should calve every 12–13 months, which generally requires conception within 85–115 days postpartum. However, inadequate detection of estrus remains a major challenge in dairy herd management, leading to delayed or improperly timed insemination, reduced reproductive efficiency, and prolonged calving intervals (Cabrera, 2014).

Estrus synchronization protocols are widely used to improve reproductive management by coordinating ovarian activity and promoting more visible behavioral estrus before breeding. In many dairy production systems, particularly under practical field conditions where farmers prefer insemination after visible heat detection, protocols that enhance behavioral estrus expression may improve insemination timing and reproductive performance. Among these protocols, Heat Synch has been extensively applied in dairy cattle. This protocol begins with administration of gonadotropin-releasing hormone (GnRH) to induce ovulation or luteinization of a dominant follicle, followed approximately one week later by prostaglandin F₂α (PGF₂α) to induce luteolysis, and estradiol benzoate one day later to stimulate behavioral estrus and the preovulatory GnRH/LH surge, after which artificial insemination is typically performed based on estrus detection (Bartolome et al., 2005).

The success of the Heat Synch protocol depends largely on the ovulatory response to the first GnRH administration. For effective ovulation induction and initiation of a new follicular wave, a physiologically mature dominant follicle should be present at the time of GnRH treatment. In cows with asynchronous follicular dynamics or postpartum anestrus, the ovulatory response to GnRH may be suboptimal, potentially resulting in poorer synchronization (Bisinotto et al., 2014)

To improve ovarian status before initiation of Heat Synch, pre-synchronization strategies using GnRH or human chorionic gonadotropin (hCG), administered approximately one week before initiation of the Heat Synch protocol, have been proposed. Both hormones may induce ovulation of an existing dominant follicle and promote corpus luteum formation before the Heat Synch protocol begins, thereby increasing the likelihood that a responsive dominant follicle will be present at the first GnRH administration of Heat Synch (Keskin et al., 2010; Liu et al., 2019). In addition, hCG possesses strong LH-like activity and a longer half-life than GnRH, which may influence luteal development and progesterone concentrations during synchronization protocols under certain physiological conditions.

Reproductive physiology and hormonal responsiveness may also vary according to parity. Primiparous cows often differ from multiparous cows in metabolic demands, nutrient partitioning, ovarian dynamics, estrous expression, and reproductive performance due to the combined demands of continued body growth and lactation (Lean et al., 2023; Morales Pineyrua et al., 2018). These physiological differences may influence responses to synchronization protocols and fertility outcomes (El-Tarabany et al., 2016). However, information regarding parity-specific responses to GnRH- and hCG-based pre-synchronization protocols within Heat Synch systems remains limited.

Therefore, the present study investigated whether pre-synchronization with GnRH or hCG before initiation of the Heat Synch protocol could improve estrus expression, progesterone concentrations, and fertility outcomes in primiparous, second-parity, and multiparous Holstein dairy cows under practical dairy farm conditions. The study also aimed to evaluate whether these pre-synchronization strategies produce comparable reproductive responses across parity groups within a Heat Synch-based reproductive management system.

## 2 Material and methods

This study was conducted on 294 Holstein dairy cows housed in six industrial cattle units in East Azarbaijan Province, Iran. All units operated under the supervision of the same farm management system and followed similar nutritional, reproductive, housing, and veterinary management protocols; therefore, they were considered as a single integrated farm in the present study. All procedures were approved by the Institutional Animal Care and Use Committee of Tabriz Medical Sciences Branch, Islamic Azad University, Tabriz, Iran. Cows were handled according to standard veterinary practices, and farm owners provided informed consent for participation. All cows underwent clinical examination, including assessment of the reproductive system and udder. Cows were enrolled in the study before their first postpartum artificial insemination, at approximately 50–70 days after calving. Animals with any of the following conditions were excluded: milk fever, lameness, history of stillbirth or cesarean section, dystocia, pregnancy duration <260 days, mastitis, twin calving, body condition score <2.75, history of luteal or follicular cysts, or uterine infections. The cows were maintained under similar management conditions across farms, including bedding type, ventilation, and a total mixed ration diet with ad libitum access to feed and water.

### 2.1 Experimental groups

Cows were first grouped according to parity (primiparous, second-parity, and multiparous) and then randomly assigned within each parity group to one of three synchronization treatments: Heat Synch (HS), GnRH+HS, or hCG+HS. The experimental groups were as follows: 1- HS1: Heat Synch protocol in primiparous cows (n = 39); 2- HS2: Heat Synch protocol in second-parity cows (n = 33); 3- HS3: Heat Synch protocol in multiparous cows (n = 30); 4- GnRH+HS1: Pre-synchronization with GnRH, 7 days before Heat Synch in primiparous cows (n = 30); 5- GnRH+HS2: Pre-synchronization with GnRH, 7 days before Heat Synch in second-parity cows (n = 33); 6- GnRH+HS3: Pre-synchronization with GnRH, 7 days before Heat Synch in multiparous cows (n = 36); 7- hCG+HS1: Pre-synchronization with hCG, 7 days before Heat Synch in primiparous cows (n = 30); 8- hCG+HS2: Pre-synchronization with hCG, 7 days before Heat Synch in second-parity cows (n = 33); 9- hCG+HS3: Pre-synchronization with hCG, 7 days before Heat Synch in multiparous cows (n = 30).

### 2.2 Pre-synchronization protocols with GnRH or hCG

Cows in groups 4 to 9, after confirmation of uterine and ovarian health by ultrasonography and collection of the first blood sample, received either 25 µg alarelin acetate (GnRH agonist; Vetaroline, Aburaihan Pharma Co., Iran; 2 mL, intramuscular injection in the neck) or 3000 IU hCG (BSV BioScience, Germany; 10 mL, intramuscular injection in the neck) seven days before initiation of the Heat Synch protocol.

### 2.3 Heat synch protocol

Following confirmation of uterine and ovarian health via ultrasonography, cows underwent the Heat Synch protocol. On Day 0, cows received 25 µg alarelin acetate, a synthetic GnRH agonist (Vetaroline, Aburaihan Pharma Co., Iran; 2 mL, intramuscular injection in the neck) to induce ovulation. On Day 7, ultrasonography was repeated and a second blood sample was collected, followed by administration of 750 µg cloprostenol sodium, a synthetic PGF₂α analog (Cloprost, Nasr Pharma Co., Iran; 2 mL, intramuscular injection in the hindquarter), which was repeated on Day 8 to induce luteolysis. On Day 9, a third blood sample was collected and 1 mg estradiol benzoate (Vetastrol, Aburaihan Pharma Co., Iran; 1 mL, intramuscular injection in the neck) was administered to stimulate estrus.

**Fig. 1:**
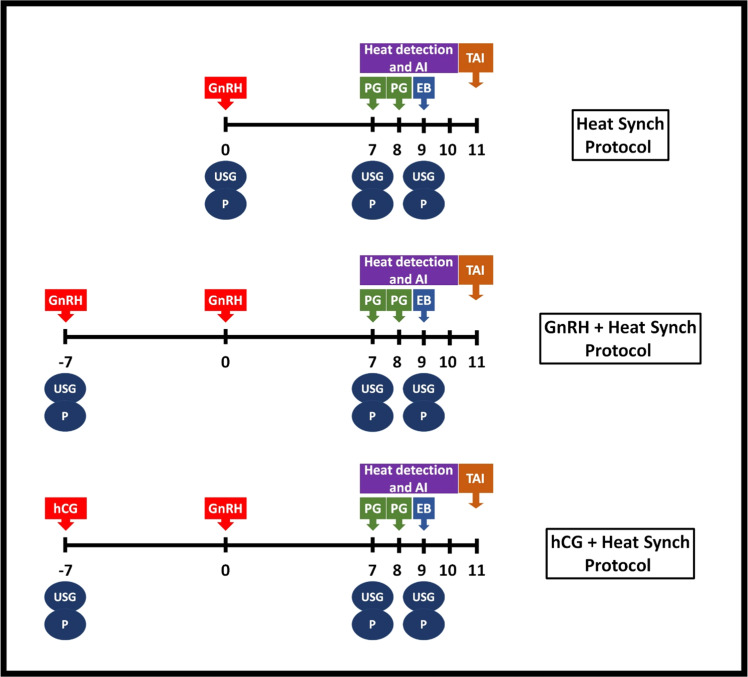
Timeline of synchronization protocols in Holstein dairy cows. HS: Heat Synch protocol (GnRH on Day 0, PGF₂α on Days 7–8, estradiol benzoate [EB] on Day 9, and artificial insemination [AI] or fixed-time AI [TAI] on Day 10). GnRH+HS and hCG+HS: pre-synchronization with GnRH or hCG on Day –7, followed by HS. USG: ultrasonography; P: serum progesterone measurement.

Estrus detection was performed from Days 7 to 11 of the protocol by visual observation for behavioral signs of estrus, including standing to be mounted, mounting activity, restlessness, vulvar swelling, and mucus discharge. Cows exhibiting behavioral estrus during this period were inseminated according to the AM–PM rule. Cows that did not exhibit estrus signs by Day 11 underwent fixed-time artificial insemination (TAI). The timelines of the synchronization protocols are illustrated in Fig. 1.

### 2.4 Pregnancy diagnosis

Pregnancy diagnosis was conducted on days 30 and 60 post-insemination using ultrasonography (SIUI CTS 800, linear probe, transrectal approach).

### 2.5 Serum progesterone level determination

Blood samples were centrifuged at 3000 rpm for 15 min, and the separated serum was stored until analysis. Serum progesterone concentrations were measured using a commercial kit (Progesterone AccuBind ELISA, Monobind Inc., USA), following the manufacturer’s instructions.

### 2.6 Statistical analysis

Data were analyzed and visualized using GraphPad Prism version 9 (GraphPad Software, San Diego, CA, USA). Estrus and pregnancy rates were compared among groups using the chi-square test, and 95% confidence intervals (CIs) for proportions were calculated using the Wilson method to illustrate variability in percentage data. Serum progesterone concentrations were expressed as mean ± SEM and analyzed by two-way analysis of variance (ANOVA), followed by Tukey’s post hoc test for multiple comparisons. A value of p < 0.05 was considered statistically significant.

## 3 Results

### 3.1 Effect of synchronization protocols on estrus expression and pregnancy risk across parity groups

The mean interval from calving to the start of the synchronization protocols is shown in Table 1.

**Table 1:** Interval from calving to initiation of synchronization protocols.

	Primiparous		Second-Parity		Multiparous	
	Mean	SEM	Mean	SEM	Mean	SEM
Heat Synch	57.54	1.72	68.36	6.38	65.60	2.80
GnRH + Heat Synch	50.73	2.10	50.33	2.38	50.47	1.67
hCG + Heat Synch	50.60	7.75	57.30	1.07	62.87	2.35

**Fig. 2:**
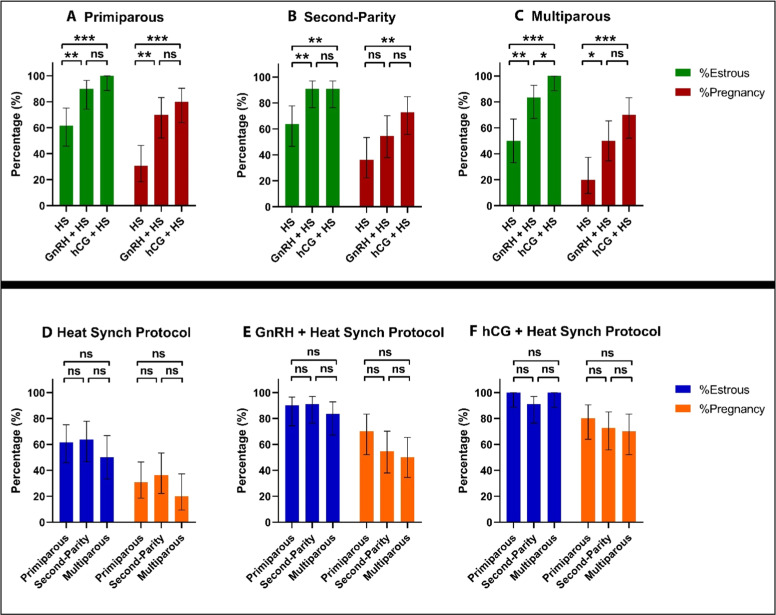
Estrus expression and pregnancy risk in Holstein dairy cows across parities under different synchronization protocols. (A–C) Rates in primiparous, second-parity, and multiparous cows, respectively. (D–F) Comparisons across parities for Heat Synch (HS), GnRH+HS, and hCG+HS protocols. Data are presented as percentages ± 95% confidence intervals (CIs) calculated from binomial distributions. Statistical comparisons were performed using the chi-square test (*p < 0.05; **p < 0.01; ***p < 0.001; ns, not significant).

In primiparous cows (Fig. 2A), the proportion of cows exhibiting estrus was significantly higher in both the GnRH+Heat Synch group (27/30; 90.0%) and the hCG+Heat Synch group (30/30; 100%) compared with the Heat Synch group alone (24/39; 61.5%) (p < 0.01 and p < 0.001, respectively). Similarly, pregnancy risk was significantly higher in the GnRH+Heat Synch group (21/30; 70.0%) and the hCG+Heat Synch group (24/30; 80.0%) than in the Heat Synch group (12/39; 30.8%) (p < 0.01 and p < 0.001, respectively). No statistically significant differences were observed between the GnRH and hCG pre-synchronization groups for either estrus expression or pregnancy risk.

In second-parity cows (Fig. 2B), the proportion of cows exhibiting estrus was significantly higher in both the GnRH+Heat Synch group (30/33; 90.9%) and the hCG+Heat Synch group (30/33; 90.9%) compared with the Heat Synch group alone (21/33; 63.6%) (p < 0.01 for both comparisons). Pregnancy risk was significantly higher in the hCG+Heat Synch group (24/33; 72.7%) compared with the Heat Synch group (12/33; 36.4%) (p < 0.01). Although pregnancy risk in the GnRH+Heat Synch group (18/33; 54.5%) was numerically higher than in the Heat Synch group, no statistically significant differences were detected between the GnRH pre-synchronization group and either the hCG pre-synchronization or Heat Synch groups.

In multiparous cows (Fig. 2C), the proportion of cows exhibiting estrus was significantly higher in both the GnRH+Heat Synch group (30/36; 83.3%) and the hCG+Heat Synch group (30/30; 100%) compared with the Heat Synch group alone (15/30; 50.0%) (p < 0.05 for GnRH+HS; p < 0.001 for hCG+HS). Similarly, pregnancy risk was significantly higher in both the GnRH+Heat Synch group (18/36; 50.0%) and the hCG+Heat Synch group (21/30; 70.0%) compared with the Heat Synch group (6/30; 20.0%) (p < 0.05 for GnRH+HS; p < 0.001 for hCG+HS). No statistically significant differences were observed between the GnRH and hCG pre-synchronization groups for either estrus expression or pregnancy risk.

### 3.2 Comparison of estrus expression and pregnancy risk among different parity groups within each synchronization protocol

When analyzing parity effects within each synchronization protocol (Fig. 2D–F), no statistically significant differences in estrus expression or pregnancy risk were observed among primiparous, second-parity, and multiparous cows within the Heat Synch, GnRH+Heat Synch, or hCG+Heat Synch protocols. The numbers and percentages of cows exhibiting estrus and/or becoming pregnant in each experimental group are summarized in Table 2.

**Table 2:** Number of cows exhibiting estrus and/or becoming pregnant in each experimental group.

	Primiparous		Second-Parity		Multiparous	
	Estrus/Total	Pregnant/Total	Estrus/Total	Pregnant/Total	Estrus/Total	Pregnant/Total
Heat Synch	24/39	12/39	21/33	12/33	15/30	6/30
GnRH + Heat Synch	27/30	21/30	30/33	18/33	30/36	18/36
hCG + Heat Synch	30/30	24/30	30/33	24/33	30/30	21/30

### 3.3 Serum progesterone concentrations

**Fig. 3:**
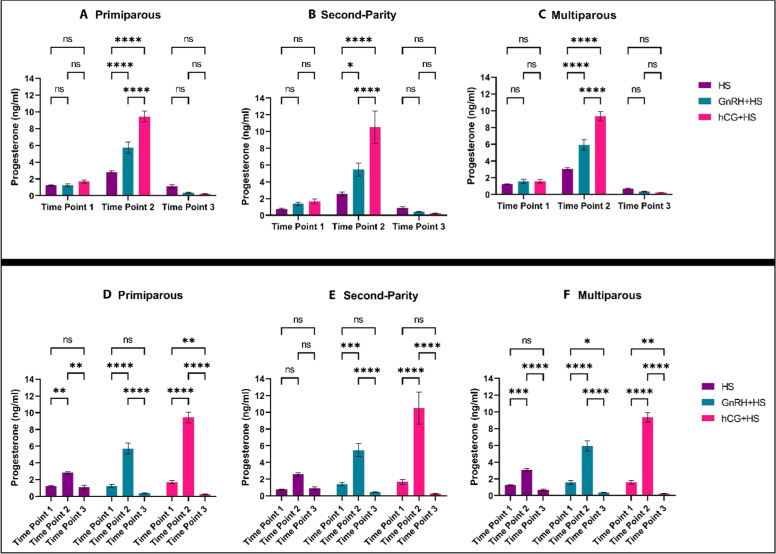
Serum progesterone concentrations at various time points in Holstein cows subjected to different synchronization protocols. Data are presented as mean ± SEM and were analyzed using two-way ANOVA followed by Tukey’s post hoc test. Time Point 1 (Day 0 in HS, Day −7 in GnRH+HS and hCG+HS), Time Point 2 (Day 7 in HS, Day 14 in GnRH+HS and hCG+HS), and Time Point 3 (Day 9 in HS, Day 16 in GnRH+HS and hCG+HS). *p < 0.05, **p < 0.01, ***p < 0.001, ****p < 0.0001, ns: not significant, HS: heat synch protocol.

At Time Point 1, the beginning of each synchronization protocol (Day 0 in the HS group and Day −7 in the GnRH+HS and hCG+HS groups), progesterone concentrations did not differ significantly among groups in primiparous, second-parity, or multiparous cows. At Time Point 2, prior to the first PGF₂α administration (Day 7 in the HS group and Day 14 in the GnRH+HS and hCG+HS groups), serum progesterone was significantly higher in both pre-synchronization protocols compared with Heat Synch alone, with the highest concentrations observed in the hCG+HS groups. By Time Point 3, prior to estradiol benzoate administration (Day 9 in the HS group and Day 16 in the GnRH+HS and hCG+HS groups), progesterone concentrations had declined in all groups, with no significant differences among treatments (Fig. 3A-C).

Within each synchronization protocol, progesterone concentrations increased from Time Point 1 to Time Point 2 and subsequently declined by Time Point 3. The rise at Time Point 2 was more pronounced in cows pre-synchronized with GnRH or hCG, particularly in the hCG+HS groups (Fig. 3 D-F).

## 4 Discussion

The present study evaluated the effect of pre-synchronization with GnRH or hCG prior to the Heat Synch protocol on fertility outcomes in Holstein cows of different parities. The results demonstrated that both pre-synchronization strategies increased estrus expression compared with Heat Synch alone, with hCG showing the most pronounced benefits. These improvements were associated with elevated progesterone concentrations at the time of PGF₂α administration, highlighting the central role of progesterone and luteal status in determining synchronization success. These improvements have practical implications for dairy herd management, as increasing pregnancy rates by 20–30% can reduce open days, lowering feed and labor costs; for example, a 10% improvement could save approximately $50–100 per cow annually by shortening calving intervals.

The success of the Heat Synch protocol depends largely on the ability of the first GnRH injection to induce ovulation of a dominant follicle and thereby initiate a new follicular wave (Bello et al., 2006). However, if a dominant follicle is absent at the time of the first GnRH administration, the protocol may fail to synchronize effectively. Pre-synchronization with GnRH or hCG helps overcome this limitation. Administration of GnRH seven days before Heat Synch induces ovulation or luteinization of existing follicles and promotes the emergence of a new follicular wave. By the time the first GnRH injection of Heat Synch is given, a responsive dominant follicle is usually present, ensuring successful ovulation and wave synchronization. Similarly, hCG induces ovulation directly through its strong LH-like activity, also triggering the development of a new follicular wave (Keskin et al., 2010; Liu et al., 2019). In both cases, pre-synchronization ensures that the ovaries are in a favorable state at the onset of Heat Synch, thereby enhancing the consistency and effectiveness of the protocol.

Pre-synchronization with GnRH or hCG may enhance luteal activity during the period preceding PGF₂α administration by inducing ovulation and promoting corpus luteum formation before initiation of the Heat Synch protocol. Ovulation induced by these treatments leads to the formation of a new CL, and in cows that already possess a functional CL, a secondary CL is established (Cunha et al., 2021). The presence of multiple CLs markedly increases circulating progesterone concentrations, which enhances endometrial receptivity and improves the likelihood of successful conception and embryo survival (Cunha et al., 2021; El Azzi et al., 2023). This mechanism was particularly evident in the hCG+HS groups, where progesterone concentrations were highest at Time Point 2. Importantly, cows with low progesterone (<1 ng/mL) at the time of PGF₂α injection typically exhibit reduced conception rates compared with cows with higher progesterone concentrations (Carvalho et al., 2014; Denicol et al., 2012). Similarly, cows lacking a CL during resynchronization protocols have been shown to have significantly lower pregnancy per insemination (Bisinotto et al., 2010; Giordano, Wiltbank, Guenther, Pawlisch et al., 2012). These findings are consistent with the results of the present study, reinforcing the importance of pre-synchronization before luteolysis.

The higher progesterone concentrations observed in the hCG groups compared with the GnRH groups may be related to the stronger luteotropic and ovulation-inducing effects of hCG, which could promote accessory corpus luteum formation before PGF₂α administration. (De Rensis et al., 2010). Unlike GnRH, which induces endogenous LH release and may have variable effects depending on pituitary responsiveness, hCG directly binds to LH receptors with a longer half-life, leading to more robust luteinization and progesterone secretion. Elevated progesterone before PGF₂α administration creates a favorable uterine environment, improves oocyte quality, and supports embryo survival (Spencer et al., 2016). Our findings regarding progesterone concentrations following hCG administration are in partial agreement with previous studies reporting enhanced luteal activity after hCG treatment under certain synchronization conditions. (Giordano, Wiltbank, Guenther, Ares et al., 2012).

It is important to note that some previous studies have not reported fertility improvements following pre-synchronization with GnRH (Alkar et al., 2011; Bruno et al., 2014; Carvalho et al., 2015). Differences between protocols, management practices, and luteolysis efficiency may account for these discrepancies. In particular, incomplete regression of CLs has been identified as a limitation of single-dose PGF₂α administration, as 10–20% of cows may experience incomplete luteolysis, reducing conception rates (Brusveen et al., 2009; Giordano et al., 2013). In the present study, the use of two PGF₂α injections likely ensured more complete CL regression, potentially contributing to the favorable outcomes observed. Indeed, Carvalho et al. reported that two PGF₂α injections 24 h apart improved luteolysis and minimized the negative effect of low progesterone levels on fertility (Carvalho et al., 2015). Thus, the combination of pre-synchronization and double PGF₂α administration may have acted synergistically to enhance fertility outcomes in this study.

One of the major findings of the present study is that primiparous, second-parity, and multiparous cows responded similarly to the synchronization protocols. This result is particularly interesting given that reproductive physiology often differs across parities. Primiparous cows typically experience a longer postpartum anestrus and slower recovery of ovarian function due to the demands of their first lactation, whereas multiparous cows often face greater metabolic stress and negative energy balance, which can impair reproduction (Gartner et al., 2019; Wathes et al., 2007). Despite these expected physiological differences among parity groups, no statistically significant differences in the effects of GnRH or hCG pre-synchronization on estrus expression or pregnancy risk were observed among the different parity groups in the present study. This may suggest that these pre-synchronization strategies could produce broadly comparable reproductive responses across parities under the conditions of this study. However, the relatively small sample size within each experimental group may have limited the statistical power to detect subtle parity-related differences or treatment × parity interactions. Therefore, these findings should be interpreted cautiously, and larger studies are needed to further evaluate parity-specific responses to pre-synchronization protocols. Additionally, the study was conducted under relatively uniform management conditions in industrial dairy farms, including similar nutritional, reproductive, and veterinary management practices. Therefore, the results may not fully reflect the greater variability in management, nutrition, estrus detection efficiency, and health monitoring that can occur in traditional dairy herds. Future research could explore the cost-effectiveness of pre-synchronization protocols, as the additional hormonal treatments may increase production costs, and evaluate their efficacy in cows with specific reproductive challenges, such as anestrus or cystic ovarian disease.

## 5 Conclusion and future directions

Pre-synchronization with GnRH or hCG prior to the Heat Synch protocol significantly improved estrus expression in healthy Holstein dairy cows across different parities, with the hCG groups showing higher progesterone concentrations, potentially associated with increased corpus luteum formation before PGF₂α administration. These findings may have practical implications for dairy herd reproductive management by improving estrus expression and pregnancy outcomes. Future research should explore the cost-effectiveness of GnRH and hCG pre-synchronization and assess their efficacy in cows with reproductive disorders, such as anestrus or cystic ovaries. Longitudinal studies are also warranted to evaluate the long-term impact of these protocols on herd productivity, calving intervals, and overall economic outcomes.

## Ethical statement

All procedures were approved by the Institutional Animal Care and Use Committee of Tabriz Medical Sciences Branch, Islamic Azad University, Tabriz, Iran. Cows were handled according to standard veterinary practices, and farm owners provided informed consent for participation.

## Declaration of generative AI and AI-assisted technologies in the manuscript preparation process

During the preparation of this work, the authors used ChatGPT (GPT-5), an AI language model developed by OpenAI and accessed via the web version, to improve the readability and language of the manuscript. After using this service, the authors reviewed and edited the content as needed and take full responsibility for the content of the publication.

## CRediT authorship contribution statement

**Pouya Navidifar:** Writing – review & editing, Writing – original draft, Supervision, Methodology, Conceptualization. **Amirali kaveh:** Writing – original draft, Methodology, Conceptualization. **Seyed Edris Hosseini:** Writing – review & editing, Writing – original draft, Methodology, Conceptualization. **Hasan keshtkar zolghadr:** Writing – original draft, Methodology. **Roxana Simiyari:** Writing – original draft, Methodology. **Saeed Taghinasab:** Writing – original draft. **Mohammad Saied Salehi:** Writing – review & editing, Writing – original draft, Supervision, Methodology, Conceptualization. **Ali Kahyani:** Writing – review & editing, Writing – original draft, Methodology, Conceptualization.

## Declaration of competing interest

The authors declare that they have no known competing financial interests or personal relationships that could have appeared to influence the work reported in this paper.
